# Anticoagulation Therapy Reduces Recurrent Stroke in Embolic Stroke of Undetermined Source Patients With Elevated Coagulation Markers or Severe Left Atrial Enlargement

**DOI:** 10.3389/fneur.2021.695378

**Published:** 2021-06-07

**Authors:** Kishan Patel, Elio Mikhael, Michael Liu, Srikant Rangaraju, Deandra Ellis, Alexander Duncan, Samir Belagaje, Trina Belair, Laura Henriquez, Fadi Nahab

**Affiliations:** ^1^Department of Neurology, Providence St. Joseph Health, Portland, OR, United States; ^2^Department of Medicine, Saint-Joseph University, Beirut, Lebanon; ^3^Department of Neurology, Mayo Clinic, Rochester, MN, United States; ^4^Department of Neurology, Emory University, Atlanta, GA, United States; ^5^Department of Neurology and Pediatrics, Emory University, Atlanta, GA, United States; ^6^Department of Pathology & Laboratory Medicine, Emory University, Atlanta, GA, United States

**Keywords:** embolic stroke of undetermined source, biomarkers, left atrial volume index, anticoagulation, cryptogenic stroke

## Abstract

**Background:** The objective of this study was to evaluate if anticoagulation therapy reduces recurrent stroke in embolic stroke of undetermined source (ESUS) patients with left atrial enlargement (LAE) or abnormal markers of coagulation and hemostatic activity (MOCHA) compared to antiplatelet therapy.

**Methods:** ESUS patients from January 1, 2017, to June 30, 2019, underwent outpatient cardiac monitoring and the MOCHA profile (serum d-dimer, prothrombin fragment 1.2, thrombin–antithrombin complex, and fibrin monomer). Anticoagulation was offered to patients with abnormal MOCHA (≥2 elevated markers) or left atrial volume index 40 mL/m^2^. Patients were evaluated for recurrent stroke or major hemorrhage at routine clinical follow-up. We compared this patient cohort (cohort 2) to a historical cohort (cohort 1) who underwent the same protocol but remained on antiplatelet therapy.

**Results:** Baseline characteristics in cohort 2 (*n* = 196; mean age = 63 ± 16 years, 59% female, 49% non-White) were similar to cohort 1 (*n* = 42) except that cohort 2 had less diabetes (43 vs. 24%, *p* = 0.01) and more tobacco use (26 vs. 43%, *p* = 0.04). Overall, 45 patients (23%) in cohort 2 initiated anticoagulation based on abnormal MOCHA or LAE. During mean follow-up of 13 ± 10 months, cohort 2 had significantly lower recurrent stroke rates than cohort 1 (14 vs. 3%, *p* = 0.009) with no major hemorrhages.

**Conclusions:** Anticoagulation therapy in a subgroup of ESUS patients with abnormal MOCHA or severe LAE may be associated with a reduced rate of recurrent stroke compared to antiplatelet therapy. A prospective, randomized study is warranted to validate these results.

## Introduction

Approximately one-third of ischemic strokes are categorized as cryptogenic strokes, and estimates suggest that approximately one-sixth of ischemic strokes meet diagnostic criteria for embolic stroke of undetermined source (ESUS) (1, 2).

Anticoagulation therapy for ESUS patients has been postulated to prevent recurrent ischemic strokes; however, empiric treatment with anticoagulation for patients with ESUS has not been deemed beneficial in large randomized controlled trials of unselected cohorts (1, 3). Secondary analysis of NAVIGATE ESUS (4) demonstrated a beneficial effect of anticoagulation in subset of patients with moderate to severe left atrial enlargement (LAE) (4). Similarly, subgroup analysis of patients enrolled in the Warfarin-Aspirin Recurrent Stroke Study trial with elevated N-terminal pro–brain natriuretic peptide showed a beneficial effect of anticoagulation in prevention of recurrent stroke (5).

Along with markers of LAE, hematological abnormalities are known to be a contributory factor for ESUS patients. The markers of coagulation and hemostatic activity (MOCHA) profile has been shown to identify patients with occult atrial fibrillation, venous thromboembolism, or undiagnosed underlying malignancy (6, 7).

The objective of this study was to evaluate if anticoagulation therapy reduces recurrent stroke in ESUS patients who have LAE on echocardiography or abnormal MOCHA profile.

## Materials and Methods

### Participants

This is a retrospective analysis of prospectively collected data. Two cohorts were compared for the analysis: a historical cohort (termed cohort 1) of ESUS patients seen at the Emory Clinic from January 1, 2015, to December 31, 2016 (7), and cohort 2 consisted of ESUS patients seen in the Emory Stroke Clinic from January 1, 2017, to June 30, 2019. All patients underwent brain imaging with a computed tomography or magnetic resonance imaging, which demonstrated a non-lacunar brain infarction during the initial evaluation. Patients with large artery atherosclerosis or occlusion, evidence of cardioembolic source after 12 lead-ECG, and >24 h cardiac telemetry on initial inpatient evaluation or echocardiography were excluded from the analysis. Patients were included if they were ≥18 years of age and had completed prolonged outpatient cardiac monitoring with either 30-day mobile cardiac outpatient telemetry and/or implantable loop recorder (Reveal LINQ; Medtronic, Minneapolis, MN). All patients underwent MOCHA testing at least 2 weeks after the ESUS as a standard part of the Emory Clinic cryptogenic stroke evaluation. Patients with an elevation of ≥2 markers were deemed abnormal based on prior studies (6, 7). Patients with patent foramen ovale (PFO) were included in the analysis. At the initial stroke clinic visit, all patients were on single antiplatelet therapy.

### Echocardiography

Standard two-dimensional and Doppler transthoracic echocardiography (TTE) was performed on a GE Vivid 7 and E9 (General Electric, Milwaukee, WI) or Philips IE 33 (Philips, Andover, MA). Left atrial echocardiographic parameters were obtained by TTE including left atrial volume index (LAVI) and left atrial diameter. For patients who did not have these parameters reported, LAE was graded by the reviewing board-certified cardiologist.

### Markers of Coagulation and Hemostatic Activity

The MOCHA profile was obtained on patients and included serum levels of d-dimer (reference value ≤ 574), prothrombin fragment 1.2 (reference value = 65–288 pmol/L), thrombin–antithrombin complex (reference value = 1.0–5.5 μg/L), and fibrin monomer (reference value < 7 μg/mL). Serum d-dimer levels were measured by high-sensitivity latex dimer assay (Instrumentation laboratories, Bedford, MA). Prothrombin fragment 1.2 and thrombin–antithrombin complexes were performed using Enzygnost ELISA kit (Siemens Healthcare, Tarrytown, New York, NY). Fibrin monomer was measured by latex immunoassay (Stago, Parsippany, NJ).

### Intervention

**Figure 1:**
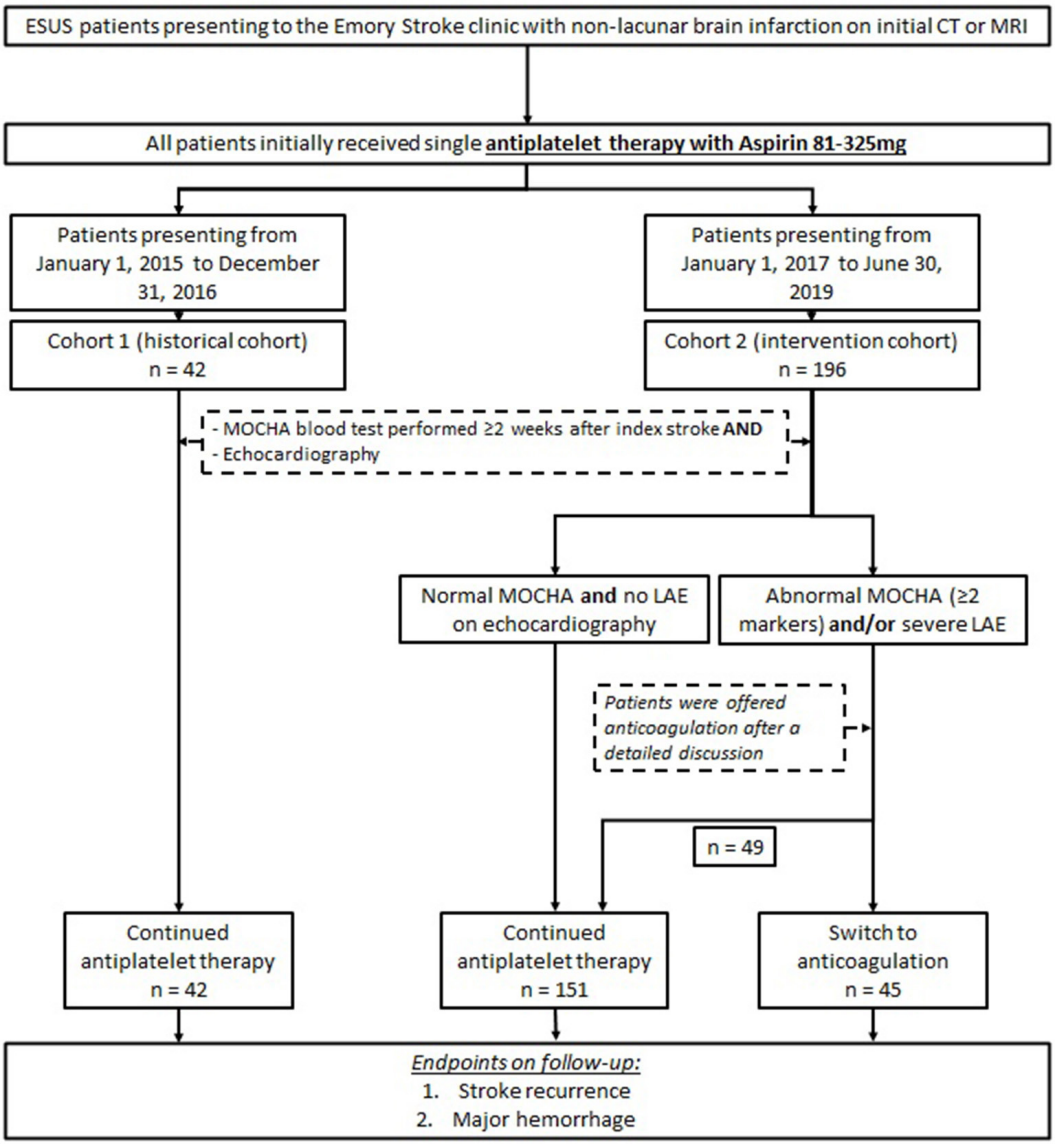
Study design.

In our two cohorts, all patients presented to the Emory Stroke Clinic on a single antiplatelet therapy with aspirin (81–325 mg daily) at first outpatient visit. Patients in cohort 2 with abnormal MOCHA panel (≥2 markers elevated) and/or evidence of LAE (LAVI ≥40 cm/m^2^) on echocardiography were offered anticoagulation therapy after a detailed discussion of available evidence and after weighing the risks of hemorrhagic complications against potential benefit of reducing recurrent stroke. Patients who elected to start anticoagulation were treated with apixaban, dabigatran, rivaroxaban, or warfarin according to provider and patient preference. Patients in cohort 2 with normal MOCHA panel and no LAE on echocardiography remained on single antiplatelet therapy. All patients in cohort 1 (historical cohort) remained on antiplatelet medication, regardless of echocardiography and MOCHA panel findings. The study design is presented in Figure 1.

### Endpoints

Patients were evaluated for the endpoints of recurrent stroke or major hemorrhage at routine clinical follow-up as previously reported (6).

### Standard Protocol Approvals, Registration, and Patient Consent

The presented study was approved by the institutional review board of Emory University.

### Statistical Analysis

The patients included in this study (cohort 2) were compared to a historical cohort (cohort 1) with abnormal MOCHA who were treated only with antiplatelet therapy during the follow-up period. The cohort 1 data were obtained from a previously published study (7). Independent *t*-tests were used for continuous variables. Categorical variables were compared with Pearson χ^2^ or Fisher exact test. Non-parametric comparison was performed using the Mann–Whitney *U*-test. Statistical analysis was performed using a combination of programs, including SPSS v25, Vassar Stats, and GraphPad. *p*-Values were calculated, and statistical significance was determined to be *p* < 0.05.

## Results

During the study period for cohort 2, 196 patients met the criteria for study inclusion. The mean age was 63 ± 16 years, 59% were female, and 49% were non-White. Compared to the historical cohort, the baseline demographic characteristics were similar (Table 1). In cohort 2, there was a lower percentage of patients with diabetes (43 vs. 24%, *p* = 0.01) but had a higher percentage of patients with any tobacco use (43 vs. 26%, *p* = 0.045). Compared to cohort 1, there was no significant difference between patients with hypertension (71 vs. 75%, *p* = 0.68) or a history of prior ischemic stroke (9.5 vs. 13%, *p* = 0.62). In cohort 2, 79 patients (40%) underwent loop recorder placement compared to 28 patients (67%) in cohort 1. All other patients underwent mobile cardiac outpatient telemetry (Table 1).

**Table 1:** Baseline characteristics.

	**Cohort 1** **(*n* = 42)**	**Cohort 2** **(*n* = 196)**	***p*-value**
Age, mean (SD), years	60 (17)	63 (16)	0.28
Female, *n* (%)	26 (62%)	116 (59%)	0.74
Race, non-White *n* (%)	24 (56%)	97 (49%)	0.37
Comorbidities, *n* (%)			
Hypertension	30 (71%)	146 (75%)	0.68
Diabetes	18 (43%)	46 (24%)	0.01
Prior ischemic stroke	4 (9.5%)	26 (13%)	0.62
Any tobacco use	11 (26%)	84 (43%)	0.045
Days from ESUS to MOCHA, median (IQR)	33 (15–57)	46(23–88)	0.30
Loop recorder, *n* (%)	28 (67%)	79 (40%)	0.002
Abnormal MOCHA (≥2), *n* (%)	23 (55%)	82 (42%)	0.13
Left atrial enlargement on echocardiography	3 (7%)	19 (10%)	0.60
Follow-up duration, mean (SD), months	13 (10)	11 (7)	0.04

Median days to MOCHA testing was 46 [interquartile range (IQR) = 23–88] days compared to 33 (IQR = 15–57) days in cohort 1. There was no significant difference in the frequency of abnormal MOCHA between cohorts with 82 patients (42%) having abnormal MOCHA (≥2 elevated markers) in cohort 2 compared to 23 patients (55%) in cohort 1. On echocardiography, 19 patients (10%) met the criteria for severe LAE (LAVI ≥40 cm/m^2^) in cohort 2, whereas 3 patients (7%) met the same criteria in cohort 1. The difference was not significant (Table 1).

In cohort 2, 45 patients (23%) were placed on anticoagulation, including 33 patients with abnormal MOCHA profile alone, 8 patients with severe LAE alone, and 4 patients with both abnormal MOCHA profile and severe LAE. In total, 37 patients (82%) received apixaban, 4 received warfarin, 2 received rivaroxaban, 1 patient received dabigatran, and 1 patient received low-molecular-weight heparin.

During mean follow-up of 13 ± 10 months, cohort 2 had significantly lower recurrent stroke rates than cohort 1 (14 vs. 3%, *p* = 0.009) with no major hemorrhages in either cohort (Table 2). One patient (2%) on anticoagulation therapy had recurrent stroke, whereas 6 (14%) in the historical cohort had a recurrence (*p* = 0.04).

**Table 2:** Results.

	**Cohort 1** **(*n* = 42)**	**Cohort 2** **(*n* = 196)**	***p*-value**
Endpoint, *n* (%)			0.009
Recurrent stroke	6 (14%)	6 (3%)	
Major hemorrhage	0 (0%)	0 (0%)	1.0

## Discussion

Our study aimed to explore the potential effects of anticoagulation in a subset of ESUS patients who have evidence of abnormal coagulation and hemostatic activity or LAE on echocardiography. Overall, we found a lower rate of recurrent stroke in a cohort of patients placed on anticoagulation therapy for abnormal MOCHA or severe LAE vs. antiplatelet therapy.

ESUS patients can be subclassified into many categories. Lattanzi et al. identified three subgroups of ESUS patients who might benefit from anticoagulation therapy (8). The first subgroup includes young males with PFO, no vascular risk factors, and posterior circulation strokes. However, a recent subgroup analysis of the RE-SPECT ESUS trial showed that anticoagulation with dabigatran compared to antiplatelet therapy did not result in reduced risk of recurrent stroke in ESUS patients with PFO (9). A second subgroup included patients with history of hypertension, diabetes mellitus, and left atrial cardiopathy with severe strokes involving multiple vascular territories. The third subgroup consisted of smokers with dyslipidemia, carotid atherosclerosis, and anterior circulation strokes (8). In addition, in a group of patients with no evidence of potential embolic source, anticoagulation seems to be superior to antiplatelet therapy (10). Nonetheless, the study is an observational one, and the findings require confirmation in a large-scale trial.

LAE on echocardiography and abnormal elevation in the MOCHA profile does identify a subgroup of ESUS patients who are at increased risk of subsequent diagnosis of atrial fibrillation and occult malignancy, respectively (7). As demonstrated in a prior study, a MOCHA profile and markers of left atrial cardiopathy may also be complementary in identifying causes of stroke in ESUS patients (6). We chose to characterize the left atrial cardiopathy by means of stratifying by LAVI on TTE. LAVI has been shown to be an independent predictor of atrial fibrillation and recurrent stroke (11). This is different than the ongoing ARCADIA trial, which is utilizing left atrial diameter on TTE but is limited by its two-dimensional measurement (12).

The present cohort was compared against a historical cohort of patients who underwent a similar protocol for diagnostic evaluation except that all patients were placed on antiplatelet therapy in cohort 1. Anticoagulation therapy offered to the subset of patients in cohort 2 who had abnormal MOCHA or severe LAE likely explains the significant reduction in recurrent stroke seen given that no patients with normal MOCHA or LAVI <40 had a recurrent stroke in cohort 2. Our stratification of treatment based on MOCHA and LAE is different from that of the NAVIGATE ESUS (3) and RE-SPECT ESUS (1) trials, which randomized ESUS patients to anticoagulation vs. antiplatelet therapy without assessment of coagulation markers or left atrial size. In these ESUS cohorts, both rivaroxaban and dabigatran were associated with an increased bleeding risk compared with antiplatelet therapy without significant reduction in stroke or systemic embolism. However, secondary analyses of NAVIGATE ESUS showed that ESUS patients with a predefined left atrial diameter of more than 4.6 cm had lower ischemic stroke rates when treated with rivaroxaban vs. aspirin (1.7 vs. 6.5%; hazard ratio = 0.26; 95% confidence interval = 0.07–0.94) (2). The reduction in ischemic stroke with anticoagulation therapy has to be balanced by the risk of hemorrhagic complication, as evidenced by another secondary analysis of the NAVIGATE ESUS trial, which found that the presence of microbleeds on baseline imaging was associated with increased stroke recurrence and increased brain hemorrhage, independent of antiplatelet or anticoagulation therapy (13).

This study has several limitations. First, it is a retrospective analysis of prospectively enrolled patients with ESUS. Second, anticoagulation treatment for ESUS patients based on coagulation abnormalities and left atrial cardiopathy remains off-label therapy. As a result of that, only 23% of cohort 2 patients elected to be placed on anticoagulation therapy. The exact treatment effect of anticoagulation cannot be directly quantified; however, future randomized studies comparing antiplatelet and anticoagulation therapy in ESUS patients based on abnormal MOCHA and/or left atrial cardiopathy will clarify optimal management for these patients. Lastly, the study was limited by a small sample size of the historical cohort.

## Conclusion

Compared to antiplatelet therapy, anticoagulation therapy may reduce recurrent strokes in the subset of patients with ESUS who have evidence of abnormal coagulation and hemostatic activity or LAE. A future study utilizing MOCHA profile and left atrial indices could also modify the diagnostic recommendations for ESUS, which can help identify occult malignancies and undiagnosed atrial fibrillation.

## Data Availability Statement

The original contributions presented in the study are included in the article/Supplementary Material, further inquiries can be directed to the corresponding author/s.

## Ethics Statement

The studies involving human participants were reviewed and approved by Institutional Review Board of Emory University. Written informed consent for participation was not required for this study in accordance with the national legislation and the institutional requirements.

## Author Contributions

KP: wrote first draft of the manuscript and contributed to manuscript revision, read, and approved the submitted version. EM: performed the statistical analysis, wrote sections of the manuscript and contributed to manuscript revision, read, and approved the submitted version. ML: organized the database, performed the statistical analysis and contributed to manuscript revision, read, and approved the submitted version. DE: organized the database, contributed to manuscript revision, read, and approved the submitted version. SR, AD, SB, TB, and LH: contributed to manuscript revision, read, and approved the submitted version. FN: organized the database, contributed to conception and design of the study, wrote sections of the manuscript, and contributed to manuscript revision, read, and approved the submitted version. All authors contributed to the article and approved the submitted version.

## Conflict of Interest

FN has a patent on the use of MOCHA to guide medical treatment in cardiovascular disease and stroke. The remaining authors declare that the research was conducted in the absence of any commercial or financial relationships that could be construed as a potential conflict of interest.

## Abbreviations

ESUS: embolic stroke of undetermined source
LAE: left atrial enlargement
MOCHA: markers of coagulation and hemostatic activation
ECG: electrocardiogram
TTE: transthoracic echocardiography
LAVI: left atrial volume index
AHA/ASA: American Heart Association/American Stroke Association
PFO: patent foramen ovale.
